# *N*-(4-Amino­pyrimidin-5-yl)-4-methyl-*N*-(4-methyl­phenyl­sulfon­yl)benzene­sulfonamide

**DOI:** 10.1107/S1600536812046442

**Published:** 2012-11-17

**Authors:** Abu Taher, Vincent J Smith

**Affiliations:** a Department of Chemistry and Polymer Science, University of Stellenbosch, Private Bag X1, Matieland 7602, South Africa

## Abstract

In the title compound, C_18_H_18_N_4_O_4_S_2_, the mean planes passing through the tosyl benzene rings form dihedral angles of 48.42 (9) and 15.1 (1)° with the amino­pyrimidine ring. In the crystal, mol­ecules associate *via* N—H⋯N and N—H⋯O hydrogen bonds, forming extended hydrogen-bonded sheets that lie parallel to the *bc* plane. The N—H⋯N hydrogen bonds propagate along the *b-*axis direction, while the N—H⋯O hydrogen bonds propagate along the *c*-axis direction.

## Related literature

For the synthesis of related sulfonamides, see: Schetty (1969 ▶); Taher & Smith (2012 ▶). For applications of ring-closing metathesis (RCM) on sulfonamide-protected allyl-containing substrates, see: Yadav *et al.* (2011 ▶); Panayides *et al.* (2007*a* ▶,*b* ▶).
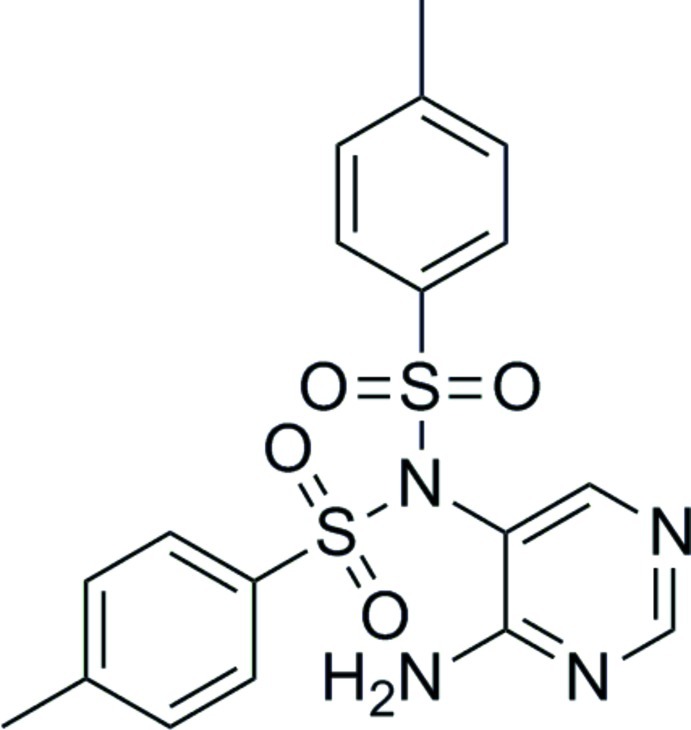


## Experimental

### 

#### Crystal data

C_18_H_18_N_4_O_4_S_2_
*M* *_r_* = 418.48
Monoclinic, 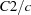

*a* = 36.559 (9) Å
*b* = 6.9044 (18) Å
*c* = 15.524 (4) Å
β = 103.852 (3)°
*V* = 3804.6 (17) Å^3^
*Z* = 8
Mo *K*α radiation
μ = 0.31 mm^−1^
*T* = 103 K
0.13 × 0.13 × 0.10 mm

#### Data collection

Bruker SMART APEX CCD diffractometer
Absorption correction: multi-scan (*SADABS*; Bruker, 2009 ▶) *T*_min_ = 0.962, *T*_max_ = 0.969
11288 measured reflections
4459 independent reflections
3156 reflections with *I* > 2σ(*I*)
*R*_int_ = 0.041

#### Refinement

*R*[*F* ^2^ > 2σ(*F* ^2^)] = 0.046
*wR*(*F* ^2^) = 0.116
*S* = 1.04
4459 reflections
254 parameters
H-atom parameters constrained
Δρ_max_ = 0.40 e Å^−3^
Δρ_min_ = −0.58 e Å^−3^

### 

Data collection: *APEX2* (Bruker, 2009 ▶); cell refinement: *SAINT* (Bruker, 2009 ▶); data reduction: *SAINT*; program(s) used to solve structure: *SHELXS97* (Sheldrick, 2008 ▶); program(s) used to refine structure: *SHELXL97* (Sheldrick, 2008 ▶); molecular graphics: *X-SEED* (Barbour, 2001 ▶; Atwood & Barbour, 2003 ▶); software used to prepare material for publication: *X-SEED*.

## Supplementary Material

Crystal structure: contains datablock(s) I, global. DOI: 10.1107/S1600536812046442/hg5262sup1.cif

Structure factors: contains datablock(s) I. DOI: 10.1107/S1600536812046442/hg5262Isup2.hkl

Supplementary material file. DOI: 10.1107/S1600536812046442/hg5262Isup3.cml

Additional supplementary materials:  crystallographic information; 3D view; checkCIF report

## Figures and Tables

**Table 1:** Hydrogen-bond geometry (Å, °)

*D*—H⋯*A*	*D*—H	H⋯*A*	*D*⋯*A*	*D*—H⋯*A*
N3—H3*A*⋯O1^i^	0.88	2.16	3.036 (3)	178
N3—H3*B*⋯N1^ii^	0.88	2.30	2.986 (3)	135

Symmetry codes: (i) 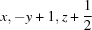; (ii) 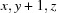.

**Fig. 1.**
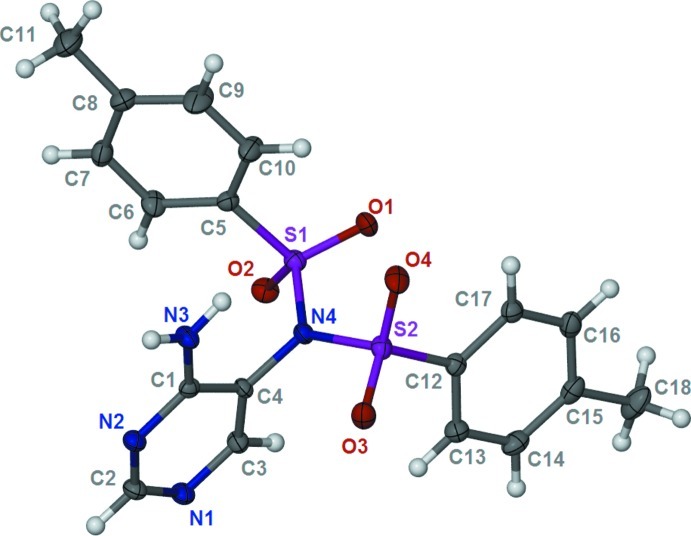
The molecular structure of the title compound showing the atomic numbering scheme - the displacement ellipsoids are shown at the 50 percent probability.

**Fig. 2.**
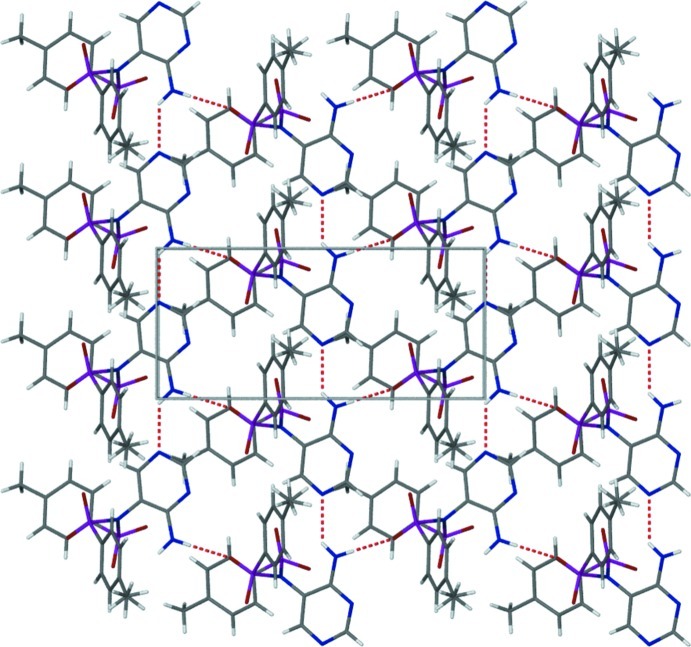
The hydrogen bonded sheet viewed along a and which runs parallel to the bc plane.

**Fig. 3.**
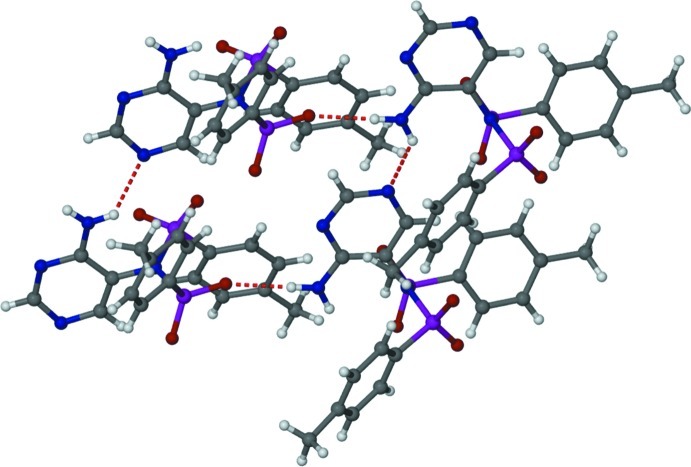
The hydrogen bond motif parallel to the bc plane.

## supplementary crystallographic information

### Comment

The *para*-toluene sulfonyl group (Ts) is frequently used as a protecting group for amines, particularly when monoalkylation of the amine is desired as the sulfonamide can then be cleaved in a subsequent step. The van Otterlo research group have successfully utilized the Ts group during their syntheses of annulated heterocycles using ring-closing metathesis (RCM) and isomerization strategies (see for example: Panayides *et al.*, 2007*a*, 2007*b*; Yadav *et al.*, 2011). In this present research the main aim was to synthesize pyrimidine-annulated heterocycles in which a 4,5-disulfonamide-protected 4,5-diaminopyrimidine was required. Surprisingly, instead of the desired 4,5-diTs compound the isomeric 5,5-disulfonamide-protected 4,5-diaminopyrimidine was obtained. It should be pointed out that according to literature it is uncommon for this type of ditosylation to occur on one amine in the presence of another amine group [see for instance Schetty (1969) and Taher *et al.* (2012)].

### Experimental

To an ice-cooled solution of 4,5-diaminopyrimidine (0.100 g, 0.908 mmol) in pyridine (10 ml), was slowly added 4-methylbenzene-1-sulfonyl chloride (0.380 g, 2.00 mmol). The mixture was then stirred at 273.15 K for 2 h. After completion of the reaction, as monitored by TLC, ice-cooled water (10 ml) was added to the reaction mixture. A white solid precipitate was formed which was collected by filteration and washed with dilute HCl (15 ml, 1 *M*) and plenty of water, after which it was dried in an oven (373.15 K). The residue was recrystallized from MeOH/CH_2_Cl_2_ to afford the product *N*-(4-aminopyrimidin-5-yl)-4-methyl-*N*-tosylbenzenesulfonamide as a colourless crystalline material (0.357 g, 94%).

### Refinement

H atoms were positioned geometrically [N—H = 0.88 Å; C—H = 0.95–0.98 Å; with *U*_iso_(H) = 1.2–1.5Ueq(N,*C*)] and constrained to ride on their parent atoms.

### Crystal data

**Table:**

C_18_H_18_N_4_O_4_S_2_	*F*(000) = 1744
*M**_r_* = 418.48	*D*_x_ = 1.461 Mg m^−^^3^
Monoclinic, *C*2/*c*	Melting point: 211 K
Hall symbol: -C 2yc	Mo *K*α radiation, λ = 0.71073 Å
*a* = 36.559 (9) Å	Cell parameters from 2317 reflections
*b* = 6.9044 (18) Å	θ = 2.3–27.0°
*c* = 15.524 (4) Å	µ = 0.31 mm^−^^1^
β = 103.852 (3)°	*T* = 103 K
*V* = 3804.6 (17) Å^3^	Prismatic, colourless
*Z* = 8	0.13 × 0.13 × 0.10 mm

### Data collection

**Table:**

Bruker SMART APEX CCD diffractometer	4459 independent reflections
Radiation source: fine-focus sealed tube, SMART APEX	3156 reflections with *I* > 2σ(*I*)
Graphite monochromator	*R*_int_ = 0.041
φ and ω scans	θ_max_ = 28.7°, θ_min_ = 2.3°
Absorption correction: multi-scan (*SADABS*; Bruker, 2009)	*h* = −48→47
*T*_min_ = 0.962, *T*_max_ = 0.969	*k* = −5→8
11288 measured reflections	*l* = −19→20

### Refinement

**Table:**

Refinement on *F*^2^	Primary atom site location: structure-invariant direct methods
Least-squares matrix: full	Secondary atom site location: difference Fourier map
*R*[*F*^2^ > 2σ(*F*^2^)] = 0.046	Hydrogen site location: inferred from neighbouring sites
*wR*(*F*^2^) = 0.116	H-atom parameters constrained
*S* = 1.04	*w* = 1/[σ^2^(*F*_o_^2^) + (0.0545*P*)^2^ + 0.5062*P*] where *P* = (*F*_o_^2^ + 2*F*_c_^2^)/3
4459 reflections	(Δ/σ)_max_ < 0.001
254 parameters	Δρ_max_ = 0.40 e Å^−^^3^
0 restraints	Δρ_min_ = −0.58 e Å^−^^3^

### Special details

**Table:**

Geometry. All e.s.d.'s (except the e.s.d. in the dihedral angle between two l.s. planes) are estimated using the full covariance matrix. The cell e.s.d.'s are taken into account individually in the estimation of e.s.d.'s in distances, angles and torsion angles; correlations between e.s.d.'s in cell parameters are only used when they are defined by crystal symmetry. An approximate (isotropic) treatment of cell e.s.d.'s is used for estimating e.s.d.'s involving l.s. planes.
Refinement. Refinement of *F*^2^ against ALL reflections. The weighted *R*-factor *wR* and goodness of fit *S* are based on *F*^2^, conventional *R*-factors *R* are based on *F*, with *F* set to zero for negative *F*^2^. The threshold expression of *F*^2^ > σ(*F*^2^) is used only for calculating *R*-factors(gt) *etc*. and is not relevant to the choice of reflections for refinement. *R*-factors based on *F*^2^ are statistically about twice as large as those based on *F*, and *R*- factors based on ALL data will be even larger.

### Fractional atomic coordinates and isotropic or equivalent isotropic displacement parameters (Å^2^)

**Table:**

	*x*	*y*	*z*	*U*_iso_*/*U*_eq_	Occ. (<1)
S1	0.089895 (16)	0.32905 (8)	0.29158 (4)	0.01860 (15)	
S2	0.169329 (16)	0.42295 (8)	0.38027 (4)	0.01945 (15)	
O1	0.10267 (4)	0.4335 (2)	0.22487 (10)	0.0232 (4)	
O2	0.07469 (5)	0.1386 (2)	0.27300 (10)	0.0237 (4)	
O3	0.19243 (4)	0.3605 (2)	0.46334 (10)	0.0249 (4)	
O4	0.16212 (5)	0.6242 (2)	0.36298 (11)	0.0260 (4)	
C4	0.12489 (6)	0.1827 (3)	0.44923 (14)	0.0165 (5)	
C1	0.11889 (6)	0.2557 (3)	0.52950 (14)	0.0169 (5)	
C12	0.18620 (6)	0.3219 (3)	0.29353 (14)	0.0186 (5)	
N3	0.11439 (5)	0.4438 (3)	0.54561 (12)	0.0206 (4)	
H3A	0.1104	0.4802	0.5969	0.025*	
H3B	0.1155	0.5310	0.5049	0.025*	
N4	0.12770 (5)	0.3096 (3)	0.37743 (11)	0.0176 (4)	
N1	0.12518 (6)	−0.1406 (3)	0.50446 (13)	0.0223 (4)	
C5	0.05758 (6)	0.4700 (3)	0.33053 (14)	0.0180 (5)	
N2	0.11685 (5)	0.1306 (3)	0.59516 (12)	0.0205 (4)	
C2	0.11992 (7)	−0.0559 (3)	0.57827 (15)	0.0225 (5)	
H2	0.1182	−0.1417	0.6250	0.027*	
C16	0.20041 (6)	0.3556 (4)	0.15198 (16)	0.0244 (5)	
H16	0.2014	0.4328	0.1019	0.029*	
C17	0.18700 (6)	0.4358 (4)	0.22029 (15)	0.0215 (5)	
H17	0.1785	0.5662	0.2171	0.026*	
C15	0.21246 (6)	0.1637 (4)	0.15541 (16)	0.0253 (5)	
C3	0.12811 (6)	−0.0140 (3)	0.44085 (15)	0.0201 (5)	
H3	0.1326	−0.0632	0.3872	0.024*	
C10	0.06049 (7)	0.6690 (4)	0.32818 (18)	0.0288 (6)	
H10	0.0806	0.7286	0.3089	0.035*	
C8	0.00387 (7)	0.6964 (4)	0.38297 (16)	0.0270 (6)	
C14	0.21133 (7)	0.0535 (4)	0.22965 (17)	0.0269 (6)	
H14	0.2195	−0.0774	0.2327	0.032*	
C6	0.02854 (7)	0.3822 (4)	0.35961 (16)	0.0253 (5)	
H6	0.0270	0.2450	0.3618	0.030*	
C13	0.19845 (7)	0.1310 (4)	0.29931 (16)	0.0243 (5)	
H13	0.1980	0.0550	0.3500	0.029*	
C7	0.00199 (7)	0.4962 (4)	0.38528 (16)	0.0252 (5)	
H7	−0.0180	0.4362	0.4049	0.030*	
C9	0.03359 (8)	0.7801 (4)	0.3544 (2)	0.0400 (7)	
H9	0.0354	0.9173	0.3530	0.048*	
C18	0.22614 (8)	0.0793 (5)	0.07884 (18)	0.0386 (7)	
H18C	0.2518	0.1247	0.0818	0.058*	
H18A	0.2094	0.1210	0.0227	0.058*	
H18B	0.2260	−0.0623	0.0824	0.058*	
C11	−0.02630 (8)	0.8178 (4)	0.4063 (2)	0.0414 (7)	
H11A	−0.0205	0.9551	0.4011	0.062*	0.50
H11B	−0.0276	0.7898	0.4674	0.062*	0.50
H11C	−0.0506	0.7878	0.3658	0.062*	0.50
H11D	−0.0453	0.7333	0.4218	0.062*	0.50
H11E	−0.0382	0.8987	0.3554	0.062*	0.50
H11F	−0.0152	0.9007	0.4571	0.062*	0.50

### Atomic displacement parameters (Å^2^)

**Table:**

	*U*^11^	*U*^22^	*U*^33^	*U*^12^	*U*^13^	*U*^23^
S1	0.0218 (3)	0.0156 (3)	0.0187 (3)	0.0002 (2)	0.0055 (2)	−0.0004 (2)
S2	0.0223 (3)	0.0153 (3)	0.0224 (3)	−0.0022 (2)	0.0086 (2)	−0.0016 (2)
O1	0.0266 (9)	0.0251 (9)	0.0195 (8)	0.0010 (7)	0.0088 (7)	0.0048 (7)
O2	0.0286 (9)	0.0155 (9)	0.0260 (9)	−0.0020 (7)	0.0048 (7)	−0.0040 (7)
O3	0.0234 (9)	0.0292 (10)	0.0221 (8)	−0.0024 (7)	0.0051 (7)	−0.0005 (7)
O4	0.0338 (10)	0.0132 (8)	0.0352 (9)	−0.0024 (7)	0.0163 (8)	−0.0023 (7)
C4	0.0196 (11)	0.0143 (11)	0.0168 (10)	0.0000 (9)	0.0067 (9)	0.0035 (9)
C1	0.0139 (10)	0.0157 (11)	0.0207 (11)	−0.0004 (9)	0.0033 (9)	0.0005 (9)
C12	0.0182 (11)	0.0169 (12)	0.0210 (11)	−0.0024 (9)	0.0054 (9)	−0.0012 (9)
N3	0.0313 (11)	0.0127 (10)	0.0196 (9)	0.0014 (8)	0.0100 (8)	−0.0006 (8)
N4	0.0199 (10)	0.0157 (10)	0.0182 (9)	−0.0003 (8)	0.0064 (8)	0.0017 (8)
N1	0.0280 (11)	0.0151 (10)	0.0255 (10)	0.0034 (8)	0.0098 (9)	0.0028 (8)
C5	0.0164 (11)	0.0180 (12)	0.0188 (11)	0.0013 (9)	0.0028 (9)	0.0005 (9)
N2	0.0238 (10)	0.0153 (10)	0.0233 (10)	0.0012 (8)	0.0071 (8)	0.0026 (8)
C2	0.0265 (13)	0.0178 (12)	0.0242 (12)	0.0033 (10)	0.0081 (10)	0.0046 (10)
C16	0.0192 (12)	0.0326 (14)	0.0217 (12)	−0.0015 (10)	0.0055 (10)	0.0009 (10)
C17	0.0194 (12)	0.0204 (12)	0.0253 (12)	−0.0009 (10)	0.0067 (10)	0.0008 (10)
C15	0.0163 (11)	0.0343 (15)	0.0252 (12)	0.0001 (11)	0.0048 (10)	−0.0075 (11)
C3	0.0234 (12)	0.0177 (12)	0.0209 (11)	0.0011 (10)	0.0084 (10)	0.0003 (9)
C10	0.0248 (13)	0.0190 (13)	0.0458 (15)	0.0006 (10)	0.0145 (12)	0.0026 (11)
C8	0.0241 (13)	0.0261 (14)	0.0316 (13)	0.0073 (11)	0.0082 (11)	0.0027 (11)
C14	0.0233 (13)	0.0216 (13)	0.0362 (14)	0.0042 (10)	0.0080 (11)	−0.0040 (11)
C6	0.0304 (14)	0.0163 (12)	0.0315 (13)	−0.0025 (10)	0.0120 (11)	−0.0014 (10)
C13	0.0245 (13)	0.0225 (13)	0.0271 (12)	0.0037 (10)	0.0083 (11)	0.0023 (10)
C7	0.0214 (12)	0.0279 (14)	0.0285 (13)	−0.0011 (10)	0.0102 (10)	0.0004 (11)
C9	0.0406 (17)	0.0145 (13)	0.072 (2)	0.0056 (12)	0.0268 (16)	0.0037 (13)
C18	0.0290 (15)	0.057 (2)	0.0314 (14)	0.0093 (14)	0.0101 (12)	−0.0127 (14)
C11	0.0361 (16)	0.0362 (17)	0.0574 (19)	0.0130 (13)	0.0223 (15)	0.0070 (14)

### Geometric parameters (Å, º)

**Table:**

S1—O1	1.4291 (16)	C17—H17	0.9500
S1—O2	1.4299 (17)	C15—C14	1.390 (3)
S1—N4	1.6800 (19)	C15—C18	1.512 (3)
S1—C5	1.747 (2)	C3—H3	0.9500
S2—O4	1.4275 (17)	C10—C9	1.383 (3)
S2—O3	1.4284 (17)	C10—H10	0.9500
S2—N4	1.703 (2)	C8—C7	1.385 (3)
S2—C12	1.755 (2)	C8—C9	1.393 (4)
C4—C3	1.372 (3)	C8—C11	1.497 (3)
C4—C1	1.409 (3)	C14—C13	1.386 (3)
C4—N4	1.441 (3)	C14—H14	0.9500
C1—N3	1.340 (3)	C6—C7	1.381 (3)
C1—N2	1.352 (3)	C6—H6	0.9500
C12—C13	1.388 (3)	C13—H13	0.9500
C12—C17	1.388 (3)	C7—H7	0.9500
N3—H3A	0.8800	C9—H9	0.9500
N3—H3B	0.8800	C18—H18C	0.9800
N1—C2	1.340 (3)	C18—H18A	0.9800
N1—C3	1.342 (3)	C18—H18B	0.9800
C5—C10	1.379 (3)	C11—H11A	0.9800
C5—C6	1.389 (3)	C11—H11B	0.9800
N2—C2	1.324 (3)	C11—H11C	0.9800
C2—H2	0.9500	C11—H11D	0.9800
C16—C17	1.386 (3)	C11—H11E	0.9800
C16—C15	1.393 (3)	C11—H11F	0.9800
C16—H16	0.9500		
			
O1—S1—O2	119.77 (10)	C5—C10—H10	120.7
O1—S1—N4	105.41 (9)	C9—C10—H10	120.7
O2—S1—N4	107.02 (10)	C7—C8—C9	118.0 (2)
O1—S1—C5	109.58 (10)	C7—C8—C11	120.5 (2)
O2—S1—C5	108.65 (11)	C9—C8—C11	121.4 (2)
N4—S1—C5	105.43 (10)	C13—C14—C15	121.2 (2)
O4—S2—O3	120.40 (10)	C13—C14—H14	119.4
O4—S2—N4	108.59 (10)	C15—C14—H14	119.4
O3—S2—N4	102.50 (9)	C7—C6—C5	119.3 (2)
O4—S2—C12	109.01 (11)	C7—C6—H6	120.3
O3—S2—C12	109.41 (10)	C5—C6—H6	120.3
N4—S2—C12	105.89 (10)	C14—C13—C12	118.7 (2)
C3—C4—C1	118.2 (2)	C14—C13—H13	120.6
C3—C4—N4	120.34 (19)	C12—C13—H13	120.6
C1—C4—N4	121.43 (19)	C6—C7—C8	121.3 (2)
N3—C1—N2	116.58 (19)	C6—C7—H7	119.4
N3—C1—C4	124.3 (2)	C8—C7—H7	119.4
N2—C1—C4	119.1 (2)	C10—C9—C8	121.8 (2)
C13—C12—C17	121.4 (2)	C10—C9—H9	119.1
C13—C12—S2	119.69 (18)	C8—C9—H9	119.1
C17—C12—S2	118.92 (18)	C15—C18—H18C	109.5
C1—N3—H3A	120.0	C15—C18—H18A	109.5
C1—N3—H3B	120.0	H18C—C18—H18A	109.5
H3A—N3—H3B	120.0	C15—C18—H18B	109.5
C4—N4—S1	117.70 (15)	H18C—C18—H18B	109.5
C4—N4—S2	119.23 (15)	H18A—C18—H18B	109.5
S1—N4—S2	122.98 (11)	C8—C11—H11A	109.5
C2—N1—C3	113.4 (2)	C8—C11—H11B	109.5
C10—C5—C6	120.9 (2)	H11A—C11—H11B	109.5
C10—C5—S1	118.89 (18)	C8—C11—H11C	109.5
C6—C5—S1	120.14 (18)	H11A—C11—H11C	109.5
C2—N2—C1	116.72 (19)	H11B—C11—H11C	109.5
N2—C2—N1	129.0 (2)	C8—C11—H11D	109.5
N2—C2—H2	115.5	H11A—C11—H11D	141.1
N1—C2—H2	115.5	H11B—C11—H11D	56.3
C17—C16—C15	121.1 (2)	H11C—C11—H11D	56.3
C17—C16—H16	119.4	C8—C11—H11E	109.5
C15—C16—H16	119.4	H11A—C11—H11E	56.3
C16—C17—C12	118.8 (2)	H11B—C11—H11E	141.1
C16—C17—H17	120.6	H11C—C11—H11E	56.3
C12—C17—H17	120.6	H11D—C11—H11E	109.5
C14—C15—C16	118.7 (2)	C8—C11—H11F	109.5
C14—C15—C18	121.4 (2)	H11A—C11—H11F	56.3
C16—C15—C18	119.8 (2)	H11B—C11—H11F	56.3
N1—C3—C4	123.4 (2)	H11C—C11—H11F	141.1
N1—C3—H3	118.3	H11D—C11—H11F	109.5
C4—C3—H3	118.3	H11E—C11—H11F	109.5
C5—C10—C9	118.7 (2)		
			
C3—C4—C1—N3	−178.4 (2)	O2—S1—C5—C6	16.1 (2)
N4—C4—C1—N3	1.7 (3)	N4—S1—C5—C6	−98.4 (2)
C3—C4—C1—N2	0.5 (3)	N3—C1—N2—C2	177.6 (2)
N4—C4—C1—N2	−179.33 (19)	C4—C1—N2—C2	−1.4 (3)
O4—S2—C12—C13	174.23 (18)	C1—N2—C2—N1	0.6 (4)
O3—S2—C12—C13	40.7 (2)	C3—N1—C2—N2	1.0 (4)
N4—S2—C12—C13	−69.1 (2)	C15—C16—C17—C12	0.9 (3)
O4—S2—C12—C17	−5.7 (2)	C13—C12—C17—C16	−0.1 (3)
O3—S2—C12—C17	−139.28 (18)	S2—C12—C17—C16	179.89 (17)
N4—S2—C12—C17	110.92 (19)	C17—C16—C15—C14	−0.9 (3)
C3—C4—N4—S1	78.1 (2)	C17—C16—C15—C18	178.7 (2)
C1—C4—N4—S1	−102.1 (2)	C2—N1—C3—C4	−1.9 (3)
C3—C4—N4—S2	−98.7 (2)	C1—C4—C3—N1	1.2 (3)
C1—C4—N4—S2	81.1 (2)	N4—C4—C3—N1	−178.9 (2)
O1—S1—N4—C4	−169.87 (15)	C6—C5—C10—C9	−0.8 (4)
O2—S1—N4—C4	−41.33 (18)	S1—C5—C10—C9	176.4 (2)
C5—S1—N4—C4	74.23 (18)	C16—C15—C14—C13	0.1 (4)
O1—S1—N4—S2	6.80 (15)	C18—C15—C14—C13	−179.4 (2)
O2—S1—N4—S2	135.34 (13)	C10—C5—C6—C7	0.9 (4)
C5—S1—N4—S2	−109.10 (14)	S1—C5—C6—C7	−176.23 (18)
O4—S2—N4—C4	−130.54 (16)	C15—C14—C13—C12	0.6 (4)
O3—S2—N4—C4	−2.12 (18)	C17—C12—C13—C14	−0.7 (3)
C12—S2—N4—C4	112.52 (17)	S2—C12—C13—C14	179.38 (18)
O4—S2—N4—S1	52.84 (15)	C5—C6—C7—C8	−0.3 (4)
O3—S2—N4—S1	−178.74 (12)	C9—C8—C7—C6	−0.4 (4)
C12—S2—N4—S1	−64.10 (15)	C11—C8—C7—C6	176.7 (2)
O1—S1—C5—C10	−28.6 (2)	C5—C10—C9—C8	0.0 (4)
O2—S1—C5—C10	−161.14 (19)	C7—C8—C9—C10	0.6 (4)
N4—S1—C5—C10	84.4 (2)	C11—C8—C9—C10	−176.5 (3)
O1—S1—C5—C6	148.63 (19)		

### Hydrogen-bond geometry (Å, º)

**Table:**

*D*—H···*A*	*D*—H	H···*A*	*D*···*A*	*D*—H···*A*
N3—H3*A*···O1^i^	0.88	2.16	3.036 (3)	178
N3—H3*B*···N1^ii^	0.88	2.30	2.986 (3)	135

Symmetry codes: (i) *x*, −*y*+1, *z*+1/2; (ii) *x*, *y*+1, *z*.
